# Implications of the Incidental Finding of a MYCN Amplified Adrenal Tumor: A Case Report and Update of a Pediatric Disease Diagnosed in Adults

**DOI:** 10.1155/2013/393128

**Published:** 2013-12-11

**Authors:** Anna Koumarianou, Panagiota Oikonomopoulou, Margarita Baka, Dimitrios Vlachodimitropoulos, Stylianos Argentos, Theodoros Piperos, Maria-Ioanna Christodoulou, Kakoulis Theodoulou, Theodoros Mariolis-Sapsakos

**Affiliations:** ^1^Fourth Department of Internal Medicine, Attikon University Hospital, Rimini 1, 12462 Haidari, Athens, Greece; ^2^Department of Medical Oncology, St Bartholomew's Hospital, West Smithfield, London EC1A 7BE, UK; ^3^Department of Pediatric Hematology-Oncology, “Pan. & Aglaia Kyriakou” Children's Hospital, Thivon & Livadias Street, 11527 Athens, Greece; ^4^Laboratory Forensic Medicine and Toxicology, Evgenidion Hospital University of Athens, 76th V. Sofias, 11528 Athens, Greece; ^5^Radiology Department, Attikon University Hospital, 1st Rimini Street, 12462 Athens, Greece; ^6^Department of Surgery, Evgenidion Hospital, 20th Papadiamantopoulou Street, 11528 Athens, Greece; ^7^Department of Biochemistry and Molecular Biology, University of Athens, Panepistimiopolis, 15701 Athens, Greece

## Abstract

MYCN is a well-known oncogene overexpressed in different human malignancies including neuroblastoma, rhabdomyosarcoma, medulloblastoma, astrocytoma, Wilms' tumor, and small cell lung cancer. While neuroblastoma is one of the most common childhood malignancies, in adults it is extremely rare and its treatment is based on pediatric protocols that take into consideration stage and genotypic features, such as MYCN amplification. Although neuroblastoma therapy has evolved, identification of early stage patients who need chemotherapy continues to pose a therapeutic challenge. The emerging prognostic role of MYCN phenotype of this disease is currently under investigation as it may redefine MYCN amplified subgroups. We describe an unusual case of adult neuroblastoma with MYCN amplification diagnosed incidentally and discuss possible therapeutic dilemmas.

## 1. Background

Neuroblastoma is a tumor of the sympathetic nervous system arising from neuroblasts (pluripotent sympathetic cells). It is the most common extracranial solid tumor in children, comprising approximately 8–10% of childhood cancers and up to 50% of cancer cases in newborn infants. It is characterised by marked biologic heterogeneity and variable clinical presentation and prognosis. It most typically involves the adrenal glands followed by the abdominal, thoracic, pelvic, and head and neck sympathetic ganglions, plexuses, and nerves, while the most common metastatic sites are bone marrow, liver, and lymph nodes [1].

The treatment of neuroblastoma in children is adjusted according to risk stratification based on multiple features, including age, histological characteristics, stage according to the International Neuroblastoma Staging System (INSS) [2], and molecular profile such as tumor cell DNA ploidy, MYCN, 1p, 17q, and 11q status [3].

Neuroblastoma is very rarely reported in adults who are usually treated according to pediatric protocols; the incidence rate for patients aged 30–39 years is about 0.2 cases per million. Despite limitations in the literature, most data suggest that it is characterized by a more aggressive clinical course and an overall worse outcome compared to neuroblastoma in children while MYCN is not usually amplified [4].

The authors describe a patient with a completely resected, incidentally discovered MYCN-amplified adrenal neuroblastoma, review the literature, and discuss possible faced therapeutic dilemmas.

## 2. Case Presentation

**Figure 1:**
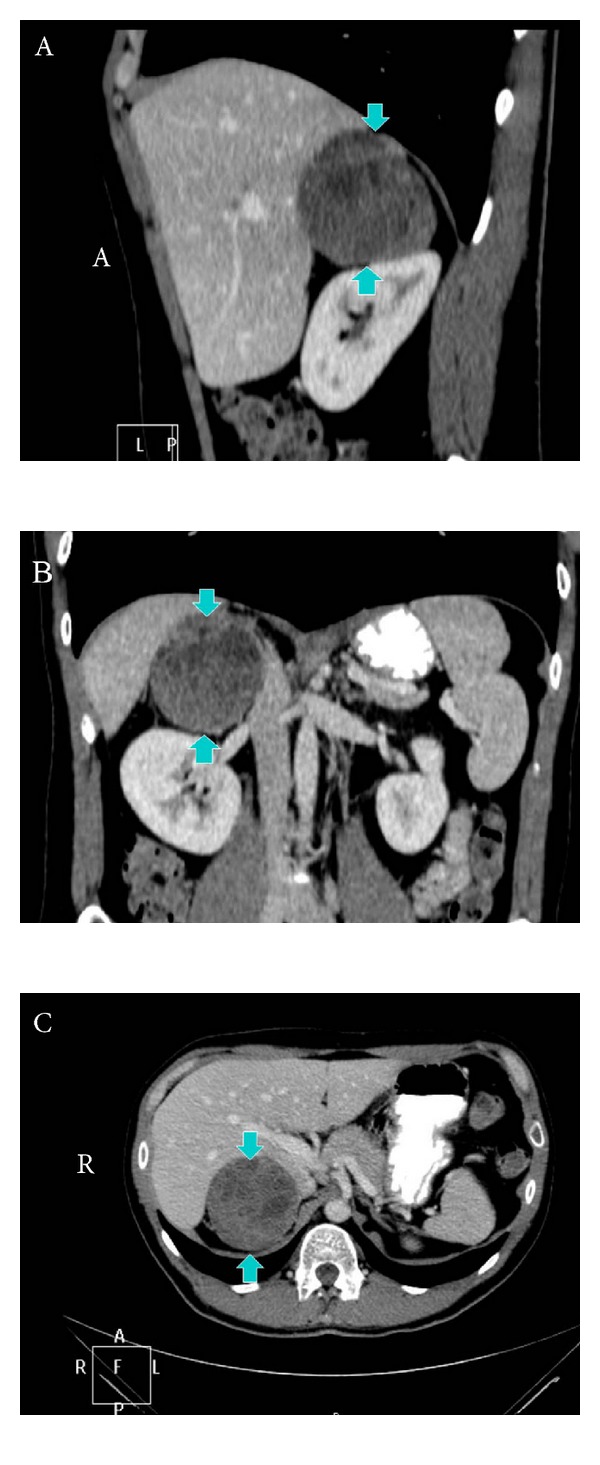
Computed tomography of the abdomen showing the right adrenal mass with malignant features, such as heterogeneous contrast enhancement. (A) sagittal plane, (B) coronal plane, and (C) axial plane (blue arrows point to the tumor).

A 30-year-old man presented to our clinic with a 7 cm solid mass of the right adrenal gland incidentally found on abdominal ultrasound during investigation of recurrent urinary infections, three and a half years ago. The patient had an unremarkable medical and family history. Physical examination and vital signs, including blood pressure, were normal. The patient's routine blood count, blood chemistries, blood serum cortisol, tumor markers including chromogranin A, NSE, and relative 24 h urine tests such as 5-hydroxyindoleacetic acid, vanillylmandelic acid (VMA), noradrenaline, dopamine, homovanillic acid (HVA), metanephrine, and potassium and sodium were also within normal limits. These normal findings together with the absence of symptoms related to hormonal secretion were suggestive of a nonfunctional tumor. On abdominal computed tomography (CT) the mass was found to exhibit malignant features, such as heterogeneous contrast enhancement (Figure 1). As adrenal glands may host metastases from other organs, a total body CT was thoroughly assessed and found negative for the presence of a different primary tumor or distant metastases. The patient was submitted to an open right adrenalectomy [5] and microscopic examination showed complete tumoral excision and no ipsilateral lymph nodal invasion.

**Figure 2:**
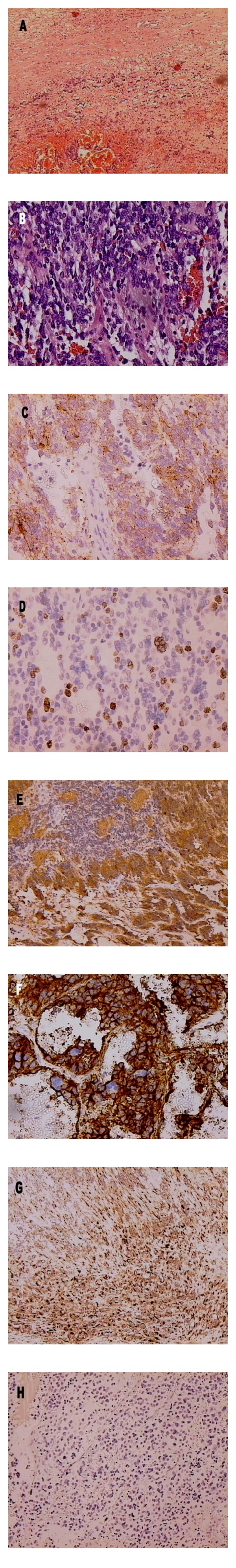
Immunohistochemical staining of the tumor cells. (A) H&E staining, ×25 magnification, (B) H&E staining, ×200 magnification (C) positive chromogranin staining, ×200 magnification, (D) positive synaptophysin staining ×200 magnification, (E) positive NSE staining, ×100 magnification, (F) positive CD56 staining, ×200 magnification, (G) positive S-100 staining, ×50 magnification, and (H) negative PanCK staining, ×100 magnification.

Histological examination of the specimen on H&E staining revealed rosettes of neoplastic cells with small size rounded cores (Figure 2). The presence of prominent nucleoli or other indicators of aggressiveness such as necrosis were not observed. On immunohistochemical staining, tumor cells were positive for chromogranin, synaptophysin, NSE, CD56, and S-100 and negative for PanCK (Figure 2). The Ki-67 labeling index was positive at 10% of the neoplastic cells. All the above findings together indicated a low grade neuroblastoma of favorable histology [6, 7].

The patient was referred to the collaborative Pediatric Hemato-Oncology Department, where a work-up usually adopted for pediatric neuroblastoma was recommended. An additionally performed bone scan and a bilateral bone marrow trephine biopsy revealed no abnormality. Chromosomal abnormalities investigated by fluorescence in situ hybridization (FISH) identified MYCN oncogene amplification (2p24) and 1p36 chromosomal deletion whereas 17q and 11q were normal [8]. The patient was stratified as stage I according to INSS. Based on at the time active International Society of Pediatric Oncology Europe Neuroblastoma (SIOPEN) Protocol LNESG2 (Localised Neuroblastoma European Study Group 2), all stage I neuroblastomas regardless of age and molecular features were assigned to frequent follow-up without any further treatment. The patient was followed up with four monthly urine VMA, dopamine, and HVA and abdominal ultrasound until three years later when he relapsed at the bed of the tumor with liver metastases and was started on first line chemotherapy according to the currently active SIOPEN protocol.

## 3. Discussion

In neuroblastoma, the choice of the appropriate treatment is based on multiple clinical and biological variables, such as disease stage and molecular features, which are usually in concordance. For instance, amplification of MYCN has been associated with advanced stage; both these features are independently correlated with adverse outcome in children [9]. Additionally, chromosomal abnormality del (1p36) is associated with age older than 1 year, advanced clinical stage, and MYCN amplification; all these characteristics predict an unfavorable outcome [10]. The role of MYCN amplification and 1p36 deletion in stage I disease remains controversial even in children [11]. Interestingly, our patient is a rare case, in which there is a lack of concordance among prognostic variables, such as low stage and genomic alterations, including MYCN amplification and 1p deletion; thus, choice of optimal treatment represents a great challenge.

Several retrospective studies have assessed the issue of controversial prognostic factors in neuroblastoma. In the Pediatric Oncology Group (POG) study of 850 children with localized neuroblastoma, only six had MYCN-amplified tumors; three remained disease free after surgical therapy, and three experienced recurrences [12]. In a subsequent POG study, only 11 of 329 children with POG stage A disease had MYCN amplified tumors; four of them never experienced recurrent disease, four relapsed and survived with additional therapy, while three died of disease [13]. A recent INRG study including 2660 patients with known MYCN status showed that patients with MYCN-amplified, low-stage tumors had less favorable event-free survival (EFS) and overall survival (OS) than patients with nonamplified tumors (53% ± 8% and 72% ± 7% versus 90% ± 1% and 98% ± 1%, respectively) [11].

Based on these latter data and the establishment of INRG classification system, MYCN amplification was upgraded to high risk [14] and chemotherapy in early stage MYCN amplified neuroblastoma patients is now being considered in a Phase III study, conducted by SIOPEN, which evaluates the benefit of 6 cycles of chemotherapy (carboplatin, etoposide, vincristine, cyclophosphamide, and doxorubicin) combined with local radiotherapy followed by 6 cycles of cis-retinoic acid.

More recently, the discrepancy between genotype (MYCN amplification detected by FISH) and phenotype (MYCN protein levels) has been described in cases of MYCN amplified neuroblastomas with better prognosis [15–18]. In the paper by Suganuma et al., the absence of prominent nucleoli, as in our patient, signified absence of MYCN RNA synthesis and was correlated with a favorable prognosis among MYCN amplified tumors [18]. As a result of the above, the discrepant genotype-phenotype may indicate that MYCN amplification does not automatically equate to protein overexpression and poor prognosis and as such may not be a valid factor for stratification of these patients in clinical trials.

Patients with MYCN positive metastatic disease may have an adverse outcome and represent a great challenge for oncologists in this field. Novel therapies targeting overexpressed proteins associated with tumor progression, such as ALK, PI3K, mTOR, Aurora, and tyrosine kinases are promising [19, 20].

## 4. Conclusions

Currently, it remains a challenge to distinguish which early stage neuroblastoma patients need chemotherapy. It is foreseen that genomic combined with proteomic analysis will be tested in prospective studies for patient stratification into different prognostic and therapeutic subgroups. Most importantly, as the biological course of neuroblastoma may differ in older patients the role of chemotherapy in this group needs to be reestablished.
